# Emerging Applications of Triazole Antifungal Drugs

**DOI:** 10.3390/ijms27020817

**Published:** 2026-01-14

**Authors:** Luiz Ricardo Soldi, Ana Paula de Lima Oliveira, Marcelo José Barbosa Silva

**Affiliations:** 1 Institute of Biomedical Sciences, Federal University of Uberlândia, Uberlândia 38405-318, MG, Brazil; 2 Faculty of Odontology, Federal University of Uberlândia, Uberlândia 38405-318, MG, Brazil; aplimaoliveira@yahoo.com.br; 3 Tumor Biomarkers and Osteoimmunology Laboratory, Av. Pará, 1720, Block 6T, Room 07, District Umuarama, Uberlândia 38400-000, MG, Brazil; majbsilva@gmail.com

**Keywords:** antifungal, triazole, chemotherapy, fungal infection, prophylaxis

## Abstract

Patients with leukemia are at heightened risk for invasive fungal infections (IFIs) due to profound immunosuppression caused by both the malignancy and its treatment. Chemotherapy-induced neutropenia, mucosal barrier disruption, and impaired innate and adaptive immune responses create a highly permissive environment for opportunistic fungal pathogens. Antifungal prophylaxis, particularly in acute myeloid leukemia (AML), has become a cornerstone in reducing IFI-related morbidity and mortality. This review outlines the immunopathogenic mechanisms underlying susceptibility to IFI and discusses current evidence on the optimal timing and therapeutic strategies for antifungal intervention. The clinical utility of key antifungal agents, namely, posaconazole, isavuconazole, and voriconazole, is critically evaluated. We also examine the potential role of emerging agents such as opelconazole, which enables targeted pulmonary delivery and prolonged epithelial retention, representing a promising approach to IFI prevention. Drug-specific considerations, including pharmacokinetics, drug–drug interactions, toxicity profiles, and cost-effectiveness, are analyzed in the context of clinical decision-making. Finally, we emphasize the importance of tailoring antifungal strategies based on leukemia subtype, immunosuppressive status, and individual patient factors to optimize outcomes and support antifungal stewardship in hematologic malignancies.

## 1. Introduction

Patients with leukemia represent one of the highest-risk groups for invasive fungal infections (IFIs), driven by the profound immunosuppression inherent to the disease and its treatment [1,2]. Chemotherapy-induced neutropenia, mucosal barrier disruption, and impaired cellular immunity create a perfect storm for the development of opportunistic fungal pathogens [3,4]. Antifungal prophylaxis has become a cornerstone of care in this population, as it significantly reduces the morbidity and mortality associated with IFI [5]. The type of leukemia, treatment intensity, and duration of immunosuppression influence the risk of IFI in patients with leukemia. In AML, prolonged neutropenia and aggressive induction chemotherapy increase vulnerability to fungal infections, particularly *Aspergillus* and *Candida* [2,6,7]. While the risk of IFI is lower in acute lymphoblastic leukemia (ALL) than in AML, it remains significant during intense chemotherapy or corticosteroid therapy. In chronic leukemias, the risk is lower than that in acute leukemias, but it increases with advanced disease stages or immunosuppressive treatments. To manage IFI, triazole drugs are used to combat confirmed infections in these patients. These drugs act primarily through inhibition of lanosterol 14-alpha-demethylase, a key fungal enzyme, which creates a weakened fungal membrane that ultimately leads to damage and death. The use of these drugs in cancer patients is limited by the location of the treatment center (with some drugs inaccessible due to pricing or transportation) [8], possible drug–drug interactions [9], and drug choice dictated by outdated treatment protocols (such as those recommending fluconazole in centers where resistant fungal strains exist) [10], which is often ultimately decided by the physician, a decision which requires a thorough analysis of patient conditions and pharmacological characteristics of the drug of choice. Although some triazole drugs have been in use for decades, such as first-generation fluconazole and itraconazole, the use of some in clinical settings has advanced from exclusive use in treatment of confirmed cases to empiric and prophylactic use [11]. Likewise, these same drugs have been further developed for use with new administration routes, such as nebulized formulas. The development of new triazole hybrid drugs, with a triazole ring in its composition, has also advanced, with potential antifungal and alternative effects, such as antitumor capabilities. Therefore, this review compiles information on the triazole drugs currently in use that have recently been tested as prophylactics in patients with hematologic cancers, opelconazole, a new antifungal under development, and triazole hybrids that have demonstrated therapeutic potential.

## 2. Immunosuppressive Effects of Leukemia Treatment on Antifungal Defense

The immunosuppressive effects of leukemia treatment significantly impair the host’s ability to mount an effective immune response against fungal pathogens. Control of fungal infections is mediated by the innate immune system, with macrophages, neutrophils, and eosinophils leading through phagocytosis, clustering/swarming, ROS production, hyphal folding, interleukin production, and inflammation promotion [12,13,14,15], and by the adaptive immune system, with pattern and antigen recognition by dendritic cells, and T and B cell subset activity. Recognition through receptors present in these immune subsets varies according to which cell and pathogen interact. For example, a mechanism occurs due to *Candida albicans* expressing beta-glucan/zymosan molecules recognized by Dectin-1 and TLR receptors in T cells, which activate a RAS/RAF1 signaling pathway, leading to increased production of IL-6, IL-12, and IL-23 [16], which stimulates the immune system to destroy invading agents. For the immune system to properly recognize and act upon these stimuli, the cells responsible must be present and capable of activity. Due to the immunosupressive effect of the tumor microenvironment, immune cells present in the bone marrow and other organs may suffer increased expression of inhibitory markers like PDL-1 and CTLA-4, leading to lower immune activity and higher cell death. In addition, the treatment for this disease in the form of chemotherapy and adjunct immunosuppressive agents sucppress both innate and adaptive immunity, two key arms of host defense critical to preventing opportunistic fungal infections, particularly those caused by *Candida* and *Aspergillus*, as well as *Rhizopus*, *Mucor*, and *Lichtheimia* species, as recognition through receptors is compromised and the overall immune response is inhibited.

Chemotherapeutic regimens alter the function and viability of innate immune cells, such as neutrophils and macrophages, which are essential for the recognition, phagocytosis, and clearance of fungal organisms [17]. The impairment of these innate immune cells leads to increased immune evasion of fungal pathogens, as they more easily avoid phagocytosis and macrophage/neutrophil swarming, while simultaneously decreasing overall inflammation and cell recruitment. For example, immunosuppressive drugs such as cyclosporine A and methylprednisolone alter the proliferation and cytotoxic activity of natural killer cells (NK), further diminishing antifungal defenses [18,19], limiting the bridge between innate and adaptive immune responses.

In addition to innate immune dysfunction, pharmacological therapy for leukemia also disrupts adaptive immune responses. T-cell immunity is particularly affected, with CD8^+^ cytotoxic T cells and CD4^+^ helper T cells exhibiting reduced functionality and viability [20]. For example, the standard 7+3 induction regimen for AML, which comprises seven days of cytarabine and three days of an anthracycline, has been shown to compromise T cell survival and cytokine production even at low doses [21]. Cytarabine-induced lymphocyte suppression, in combination with corticosteroids and calcineurin inhibitors, impairs the activity of Th1 and Th17 subsets, which are crucial for defense against *Aspergillus fumigatus* [22]. This leads to the host being unable to resist opportunistic infections, which results in increased occurrences of pulmonary or systematic IFI and other types of infections.

Although immunosuppressive therapy is essential for the management of leukemia, its impact on host immunity markedly increases susceptibility to IFI. These infections in vulnerable patients can be lethal and are one of the most common reasons for patient mortality or persistent chronic side effects after treatment, both physical (for example: pain, fatigue, neuropathy, heart or lung problems) [23] and psychological (anxiety, depression, cognitive changes, post-traumatic stress) [24]. This underscores the need for vigilant monitoring and tailored antifungal prophylaxis during and after treatment.

## 3. Approaches to Antifungal Treatment Timing and Selection

Early and appropriate antifungal intervention is a critical determinant of clinical outcomes in high-risk hematologic populations, particularly individuals undergoing chemotherapy for acute leukemia [4]. Quantitative evidence indicates that timely initiation, whether empirical or pre-emptive, modifies antifungal exposure, treatment duration, infection detection rates, and ultimately clinical outcomes.

High-resolution computed tomography (HRCT) has demonstrated measurable impact on treatment timing. In a real-world study from the Royal Marsden NHS Foundation Trust, HRCT-guided initiation reduced unnecessary antifungal exposure without affecting mortality or treatment success [25]. Reducing inappropriate exposure is clinically meaningful because earlier confirmation (radiologic or laboratory) shortens diagnostic uncertainty and accelerates targeted therapy. Although laboratory confirmation remains essential for definitive diagnosis, physicians may initiate empiric treatment when clinical features suggest possible or probable invasive fungal infection (IFI), especially in neutropenic patients.Although laboratorial confirmations are necessary for targeted treatments, it is possible for physicians to infer possible/probable infections through patient symptoms and characteristics, and initiate empiric treatments.

Empirical antifungal therapy remains a standard approach for persistent febrile neutropenia [26]. However, quantitative comparisons demonstrate that pre-emptive strategies, initiated only when clinical, radiologic, or biomarker evidence emerges, substantially reduce antifungal exposure without compromising survival. A meta-analysis reported a Relative Risk (RR) for antifungal exposure of 0.48 (95% CI 0.27–0.85) with pre-emptive therapy, indicating roughly a 52% reduction compared to empirical treatment [27]. In the randomized trial, 61.3% of the empirical arm received antifungals versus 39.2% in the pre-emptive arm, demonstrating a 36% relative reduction in drug use [28].

Moreover, among patients who did receive antifungals, treatment duration was consistently shorter in the pre-emptive group (4.5–8.7 days vs. 7.0–11.2 days).

Evidence is more heterogeneous regarding infection detection. In this trial, the rate of proven/probable invasive fungal disease (IFD) was 4.1% in the empirical arm vs. 19.5% in the pre-emptive arm, demonstrating increased detection when imaging and biomarkers guide therapy [29]. Quantitative synthesis across studies yielded a pooled RR for IFD detection of 1.70 (95% CI 1.12–2.57; M-H fixed-effects model) and 1.47 (95% CI 0.55–3.96; D-L random-effects model) [27]. Importantly, however, increased detection did not translate into higher mortality. Among the five studies reporting IFD-related deaths reviewed in this meta analysis, none observed significant differences between strategies (pooled M-H RR 0.85, 95% CI 0.45–1.62; D-L RR 0.82, 95% CI 0.36–1.87) [27].

Treatment duration is another crucial determinant of resistance, toxicity, and resource utilization. Evidence indicates that shorter durations, particularly for candidemia, do not worsen survival while reducing selective pressure for resistance. For invasive mold infections, treatment remains prolonged because of ongoing immunosuppression, but extended therapy is associated with increased toxicity. International guidelines recommend durations ranging from 3 to 6 months depending on clinical response, host immune recovery, and imaging findings [30,31,32]. Quantitatively, trials illustrate meaningful reductions with pre-emptive strategies. Cordonnier et al. (2009) [28] demonstrated that an an average antifungal duration was 7.0–11.2 days in the empirical group and 4.5–8.7 days in the pre-emptive group.

Among current antifungal classes, triazoles remain frontline therapies because of their broad spectrum and efficacy in hematologic malignancies. Among the existing antifungals, triazoles, whose primary function is the inhibition of ergosterol biosynthesis [33], have been highlighted as frontline therapies for IFI. Prophylaxis is frequently achieved with polyenes or azoles [11], and combination approaches, including aerosolized liposomal amphotericin B with fluconazole, are employed to maximize coverage in high-risk settings [34].

Quantitative risk stratification is essential for individualized therapy. Patients undergoing allogeneic stem-cell transplantation have markedly elevated IFI risk due to prolonged neutropenia; this risk increases further with graft-versus-host disease, rejection, or organ dysfunction [35]. Drug–drug interactions also influence agent selection. For example, the interaction between vincristine and triazoles produces neurologic and gastrointestinal toxicity [36], with significant consequences for quality of life and oncologic treatment adherence [37,38].

Although antifungal resistance remains a major concern, recent frameworks demonstrate that in hematologic cancer populations, the clinical benefits of optimized antifungal regimens outweigh the risks of adverse events and resistance development [39].

The optimal management of invasive fungal infections in high-risk hematologic patients depends on timely, individualized, and evidence-based strategies. Pre-emptive antifungal therapy, guided by clinical and diagnostic tools, reduces unnecessary drug exposure and shortens treatment duration without compromising survival, while empirical therapy remains essential in high-risk scenarios like febrile neutropenia. Risk stratification, considering factors such as immunosuppression, drug interactions, and organ dysfunction, ensures that interventions are both effective and tailored, minimizing toxicity and resistance while maximizing patient outcomes.

## 4. Current Triazole Antifungal Agents

### 4.1. Posaconazole

Posaconazole, a second-generation triazole antifungal, plays a central role in both the treatment and prophylaxis of invasive fungal diseases (IFDs), particularly in immunocompromised patients. Its broad-spectrum antifungal activity, combined with the availability of multiple formulations, including oral suspensions, delayed-release tablets, and intravenous (IV) solutions, has expanded its utility across various clinical settings [40,41].

Clinically, posaconazole is primarily used for prophylaxis in patients with AML, myelodysplastic syndromes, and in those undergoing hematopoietic cell transplantation (HCT) with graft-versus-host disease. It is also indicated for the treatment of oropharyngeal candidiasis and refractory IFDs [42]. The IV formulation allows for use in patients unable to tolerate oral medications and enables step-down therapy once clinical stability is achieved [43]. Additionally, posaconazole demonstrates synergistic effects when combined with other antifungals, such as caspofungin and amphotericin B, further broadening its therapeutic applications [44]. Posaconazole and its broad spectrum allow it to be used for treatment of several systemic mycoses such as candidemia, aspergillosis, mucormycosis, and even rarer infections such as hyalohyphomycosis and chromoblastomycosis [45].

Although considered a drug with good tolerability [5], adverse effects can occur with posaconazole use, with rates comparable to other antifungals [46]. Recent clinical trials have investigated its safety and efficacy in pediatric populations, including children and adolescents, indicating potential expansion of approved use [41,47]. In AML patients, posaconazole has shown superior prophylactic efficacy compared to fluconazole (11.3% vs. 28.3% IFI incidence) [48] and itraconazole (33% vs. 58%) [49].

Posaconazole is generally well tolerated, even with prolonged use. Most adverse effects are mild, such as nausea and vomiting [48,50], although hepatotoxicity has been reported in rare cases [42,51]. Despite its clinical benefits, challenges remain. These include drug–drug interactions, particularly due to cytochrome P450 (CYP3A4) inhibition, and variable absorption associated with oral formulations [40,41]. These interactions are of particular concern in populations co-administered antiretroviral therapy, such as people living with HIV, where altered pharmacokinetics may affect drug efficacy [52].

Another significant limitation is cost. Posaconazole’s high cost-effectiveness ratio may restrict its availability in low-resource patients [53]. Therapeutic drug monitoring is recommended in these cases, as adjusting the required dosage without improvident applications can reduce overall costs [54]. Ongoing research aims to clarify its economic feasibility. However, heterogeneity in study design, such as dosage, treatment duration, and patient populations, complicates direct comparison across trials.

### 4.2. Isavuconazole

Isavuconazole, a novel broad-spectrum triazole antifungal, has emerged as a valuable therapeutic option for the treatment of IFI, particularly in immunocompromised patients with hematologic malignancies. Approved by the FDA for the treatment of invasive aspergillosis and mucormycosis, isavuconazole has demonstrated comparable efficacy to voriconazole and liposomal amphotericin B in respective indications [25,55].

Clinical data support its use in patients with hematological disorders. In one study, isavuconazole achieved a favorable clinical response in 40% of the patients at 6 weeks and 60% at 12 weeks [25]. A multicenter case series from China reported a 75% overall response rate among patients treated for various fungal infections, including aspergillosis and mucormycosis [56]. Similarly, another study found a 67% response rate in hematologic patients, with complete resolution in 42% of cases [57]. Isavuconazole is generally well tolerated and associated with a lower incidence of adverse events compared to other antifungal agents. A recent meta-analysis confirmed significantly fewer drug-related side effects and treatment discontinuations [58]. Although liver enzyme abnormalities were reported in up to 29% of patients, these rarely required therapy discontinuation [59]. Isavuconazole is also considered safer in patients receiving QTc-prolonging medications and those on agents such as venetoclax or ruxolitinib, making it suitable for complex pharmacologic regimens in leukemia [60].

Pharmacokinetically, its extended half-life (approximately 184 h) allows for once-daily dosing, improving adherence and facilitating outpatient management [61]. While primarily indicated for treatment, isavuconazole has also shown potential as a prophylactic agent in high-risk hematologic populations, although further large-scale studies are needed to define its role in this context [62,63].

Combination therapy with other antifungals has been explored, though current data suggest no significant advantage over monotherapy [25]. The choice to initiate isavuconazole may be influenced by prior antifungal failure, intolerance to other agents, or the need for oral therapy to facilitate early hospital discharge [63]. In a review of global consensus guidelines, isavuconazole use as a first-line drug for treatment of mucormycosis, where amphotericin B is unavailable or ineffective, is recommended. However, solid evidence is lacking for more extensive use in clinical scenarios and for treatment of other mycoses [64].

Comparative studies indicate that isavuconazole offers similar efficacy with a more favorable safety profile compared to amphotericin B and voriconazole, particularly due to a lower incidence of adverse effects [65]. When compared to posaconazole, it shows equivalent effectiveness, while posing fewer risks for drug–drug interactions [66].

### 4.3. Voriconazole

Voriconazole is a widely used triazole antifungal agent that has demonstrated efficacy in the prophylaxis of IFI among patients with hematologic malignancies, particularly AML. Its use has been associated with significant reductions in IFI incidence in both pediatric and adult populations. In a study involving children with AML, voriconazole prophylaxis significantly lowered the incidence of IFI compared to no prophylaxis [67]. Similarly, in adult AML patients, an incidence rate of only 1.6% for proven or probable IFI was reported during induction chemotherapy [68]. A comparative study also showed that both voriconazole and posaconazole reduced breakthrough infections more effectively than fluconazole or itraconazole [69].

Despite its efficacy, clinical outcomes with voriconazole can vary significantly among individuals, primarily due to genetic polymorphisms affecting drug metabolism. The cytochrome P450 enzyme CYP2C19 plays a key role in voriconazole metabolism; rapid metabolizers may experience subtherapeutic plasma concentrations, reducing treatment efficacy. CYP2C19 genotyping has been used to customize dosing, leading to improved trough levels and a reduced risk of infections in patients with genetic predispositions [70,71].

Voriconazole is generally well tolerated [46,72]. The most common adverse events include transient visual disturbances and reversible liver enzyme elevations [73]. In patients with a history of pulmonary fungal disease, it has been successfully used as secondary prophylaxis with minimal toxicity, enabling completion of planned leukemia treatment [74]. Its favorable safety profile makes it a viable alternative to other azoles such as itraconazole, which is associated with a higher risk of neurotoxicity when used concomitantly with certain chemotherapeutic agents [73].

While voriconazole offers broad-spectrum antifungal coverage and is available in both oral and intravenous formulations, careful consideration of drug interactions and the need for therapeutic drug monitoring remains essential. Monitoring of plasma concentrations, for example, are shown to require optimization, as some studies have presented lower values than the therapeutic range required without dose adjustment [75]. Personalized dosing strategies, including pharmacogenetic-guided adjustments, may enhance its safety and efficacy, but additional prospective studies are needed to validate these approaches and standardize clinical protocols.

Compared to other antifungals, voriconazole has shown lower rates of IFI and fewer premature discontinuations of prophylaxis [69]. Its efficacy is comparable to posaconazole and isavuconazole in AML patients undergoing induction chemotherapy, with no significant differences observed in IFI rates [51].

### 4.4. Itraconazole

Itraconazole is an antifungal drug developed as an alternative to fluconazole due to the latter’s ineffectiveness to *Aspergillus* spp. and some *Candida* species (i.e., krusei and glabrata) [76]. As a broad-spectrum antifungal, it is capable of acting as a prophylactic displaying effectiveness against aspergillosis, candidosis, cryptococcosis, and several other mycoses.

Itraconazole is often administered as either a capsule or as an oral solution, with or without food, or intravenously, with good tolerance at short-term use in patients. Common side effects, which occur in approximately one-quarter of patients, include hepatotoxicity, nausea/vomiting, and diarrhea [77]. Although considered a drug with good tolerance [46], due to the frequency of these side effects being comparable to other antifungals, such as amphotericin B, itraconazole has been recognized to be among the most hepatotoxic and should therefore only be used when liver health of the patient is being regularly evaluated [78,79].

The use of oral itraconazole as a prophylactic has shown comparable results to voriconazole or posaconazole in pediatric patients undergoing stem cell transplant [80]. In another study, the incidence rates of IFI that occurred with fluconazole or itraconazole prophylaxis were also comparable, although posaconazole showed a lower rate of IFI and a higher overall survival [76]. Similarly to other antifungals, resistance profiles have already been observed in patients for itraconazole, such as some species of *Candida* [81]. Therefore, the minimum inhibitory concentration and current existing profiles of fungal infections in each location should be considered if a treatment is indicated with itraconazole.

Currently, an inhaled formulation is under development, aimed at enhancing pulmonary dispersion [57]. In an initial study, this novel route has resulted in the antifungal accumulating in lung tissue for extended periods, maximizing its local effect in the lungs. This is especially interesting for hematological malignancies whose treatments affect the lungs, such as bleomycin induced fibrosis occurring in lymphoma patients receiving ABVD regimens. Another variation currently under research is super-bioavailable itraconazole (SUBA-ITZ), a formulation developed to overcome limitations like absorption through a polymer matrix which helps disperse the drug, allowing for lower dosing achieving the same serum concentrations as conventional itraconazole administrations [82]. Conventional Itraconazole releases in the more acidic pH of the stomach, and precipitates in the more basic pH of the intestine, thereby limiting its potential absorption throughout the digestive system. The polymer matrix acts as a buffer to restrict the complete release in acidic pH values and solubilizes when it enters the intestine, providing greater bioavailability due to more thorough absorption of itraconazole. For treatment of endemic mycoses, SUBA-ITZ showed comparable effectiveness to conventional itraconazole, with fewer serious adverse effects [83]. SUBA-ITZ has also shown enhanced pharmacokinetic parameters, greater bioavailability and systemic drug distribution, and improved cardiac safety [84]. As SUBA-ITZ has shown such promising potential, it is possible that other future drugs, including antifungals, with poor solubilities may be altered using SUBA technology to compensate for their inefficiencies, promoting a series of more effective antifungal treatments.

Recently, some studies have indicated that itraconazole not only exhibits antifungal activity but also exerts inhibitory effects on various malignancies, via multiple pathways [85]. Itraconazole has shown inhibition through the hedgehog pathway, AKT/mTOR/S6K, reactive oxygen species (ROS), Wnt/beta-catenin pathways, and the HER2/AKT signaling pathway, thereby exerting anticancer effects [86,87]. Although itraconazole seemingly exerted antitumor effects in these studies, its specific molecular mechanisms are not fully understood, and, therefore, its future potential as an anticancer drug is still under investigation.

Table 1 summarizes the main antifungal agents used in leukemia patients.

## 5. Emerging Therapy

### 5.1. Opelconazole

**Figure 1:**
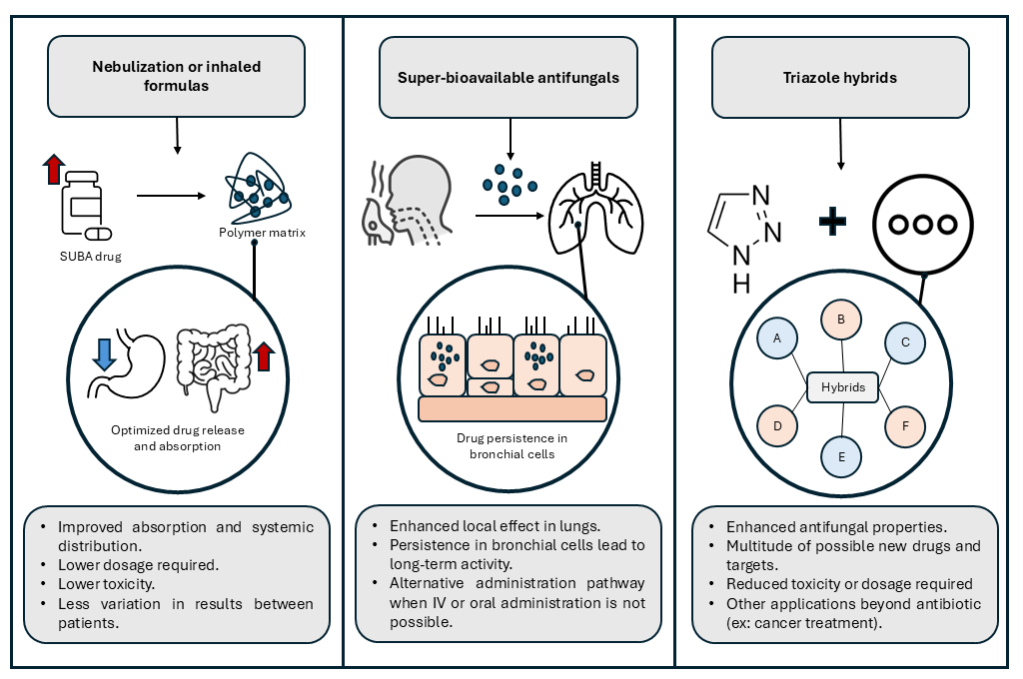
Summary of the emerging applications of antifungal drugs.

Future directions in antifungal therapy for leukemia patients involve the development of agents with novel mechanisms of action, as well as the introduction of new compounds within existing antifungal classes [88], as summarized in Figure 1. Opelconazole, currently in phase III clinical trials for the treatment of invasive pulmonary aspergillosis [89], represents a promising advance in this field.

What distinguishes Opelconazole is its mode of administration, via nebulization, which enables direct pulmonary delivery. This targeted approach is hypothesized to improve the antifungal efficacy in the lungs while reducing systemic exposure and associated toxicities such as nausea, vomiting, and hepatotoxicity [90]. Opelconazole exhibits broad-spectrum activity against several fungal pathogens, including *Aspergillus* spp., *Candida* spp., *Trichophyton rubrum*, *Cryptococcus gattii*, *C. neoformans*, *Penicillium chrysogenum*, and *Rhizopus oryzae* [91].

Although still in early development, preclinical animal studies suggest a favorable safety profile, with mild asthma-like symptoms being the only notable adverse effect reported to date [90,92,93]. Comparative efficacy data remain limited; however, initial in vitro findings indicate antifungal activity against *Candida* species that is comparable to that of established agents such as voriconazole and posaconazole [91].

A notable feature of opelconazole is its observed persistence in respiratory epithelial tissue and local immune cells [94]. If validated in human studies, this could support its use as a prophylactic agent, particularly in targeting pulmonary fungal infections. Given that the lungs are a primary site of invasive fungal disease in leukemia patients, opelconazole’s targeted delivery and potential for long-term local activity may offer a novel and effective strategy for IFI prevention in this high-risk population.

### 5.2. Triazole Hybrids

A recent trend in drug development for treatment of various diseases [95], including fungal infections, are triazole hybrids. These compounds, synthesized by conjugation with other substances, like tetrazoles [96,97] or glycosides [98], vary greatly in both structure and effect. Although some of these compounds, such as the tetrazole oteseconazole [99], possess antifungal properties against different species such as *Candida* by itself, other substances as singular compounds do not present these innate capabilities and serve mainly as a way to increase the effect of or alter the structure of existing triazoles. Although several of these compounds which use triazoles as part of their structure do not offer any antifungal property, they have been shown to possess other effects such as anti cancer properties [95].

Although a limited number of drugs with a triazole scaffold have been marketed in the last decade (ex: tazobactam, cefatrizine, and carboxyamidotriazole), testing and development of various other conjugations with triazoles are under research [100]. These marketed drugs range from antibacterial to antineoplasic treatments, each with its own unique characteristics and limitations. Among the novel compounds under development, limited in silico or in vitro assays have been performed, strictly designating these as nascent topics. In vitro and few in vivo assays of potential antifungal drugs conducted demonstrated comparable efficacy to current triazole antifungals such as fluconazole and itraconazole against *Candida* and *Aspergillus* [96,101]. Currently, these results are interesting because a comparable effectiveness to current drugs means that these new drugs may serve as key participants in the treatment of resistant strains of IFI, which are expected to develop and be seen more frequently in the coming future. Although more robust results may take several years, these triazole hybrids are nevertheless promising candidates for the future treatment of resistant strains of common fungal infections [96].

## 6. Challenges and Future Directions

Despite recent advancements in antifungal therapy for hematological malignancies, several critical challenges persist. The crux of these include the emergence of drug-resistant fungal strains, high treatment costs, significant drug–drug interactions, and limited availability of antifungal agents in low-resource settings. Furthermore, although personalized approaches, such as CYP2C19 genotyping for voriconazole dosing and individualized prophylactic strategies, show great potential, further validation through large-scale clinical studies is needed before widespread implementation. However, a serious lack of standardization occurs in several published studies, limiting comparisons between different triazole drugs, geographical regions, and treatment strategies. For example, the classification of possible, probable, and proven IFI differs between older and newer studies, and it is recommended that newer studies attune themselves to novel consensus definitions [102]. As demonstrated in meta-analysis and systematic reviews, more detailed results, with the addition of information on the therapeutic monitoring of serum dosage, would be beneficial to better understand the genuine effectiveness of each strategy [5,27,55,75].

Antifungal resistance varies between geographical regions and clinical settings, limiting the possibility of comparison of results between studies. Emerging strains resistant to conventional antifungal therapy in some regions may mask the usefulness of prophylactic strategies in others where these strains do not yet exist. Careful consideration must be taken, in conjunction with laboratorial surveillance, to determine whether current protocols must be adjusted in each setting [103]. The lack of access to specific diagnostic tests, such as imaging or antifungal susceptibility cultures, and different antifungals for treatment in low-resource settings also limit possibilities, requiring a dependency on dose management or combination therapy of available antifungals rather than targeted strategies [8,104]. Resistance to triazoles has been seen in *Candida*, *Aspergillus fumigatus* and other pathogenic fungi. Specific gene mutations have been investigated and several different mechanisms of action for resistance have been observed [105]. These fungi responsible for infections in cancer patients limit the chances of survival and, therefore, new and alternative methods of treatment are essential to keep up with novel resistance profiles.

Future research efforts should focus on the development of antifungals with novel mechanisms of action, improved drug delivery systems, and enhanced diagnostics that enable early and accurate identification of invasive fungal infections. Addressing these challenges will be essential to improving outcomes and ensuring equitable access to effective antifungal therapies across diverse patient populations.

## 7. Conclusions

IFIs represent a persistent and severe threat to leukemia patients, particularly those undergoing intensive chemotherapy and immunosuppressive therapy. The substantial immune disturbance associated with leukemia treatment demands strategic antifungal prophylaxis and therapy to reduce morbidity and mortality. Among antifungal agents, triazoles—particularly posaconazole, isavuconazole, itraconazole, and voriconazole—have demonstrated efficacy in both prophylactic and therapeutic settings, each with distinct benefits and limitations related to spectrum, safety, tolerability, drug interactions, and cost.

While posaconazole is well-established in AML patients, offering broad coverage and multiple formulations, isavuconazole emerges as a promising alternative with fewer adverse events and drug interactions. Voriconazole remains effective, but requires personalized dosing due to metabolic variability. Novel agents, such as opelconazole, show potential with innovative delivery systems that can offer organ-specific prophylaxis with reduced systemic toxicity.

Ultimately, the choice of antifungal strategy should be guided by patient-specific factors, disease severity, stage of treatment, and risk of resistance. The continued refinement of antifungal stewardship, guided by diagnostic tools, pharmacogenomics, and emerging therapies, is crucial for optimizing outcomes in this vulnerable patient population.

## Figures and Tables

**Table 1:** Summary of antifungal agents for use in Leukemia patients.

Agent	Approved Indications	Formulations	Key Advantages	Limitations/Challenges
Posaconazole	Prophylaxis and treatment of IFDs in AML, MDS, post-HCT	Oral, IV	Broad-spectrum; effective prophylaxis in AML; well tolerated	High cost; CYP3A4 interactions; variable oral absorption
Isavuconazole	Invasive aspergillosis and mucormycosis	Oral, IV	Fewer drug interactions; long half-life; better safety profile; QTc-neutral	Limited prophylactic data; rare hepatic disturbances
Voriconazole	Prophylaxis and treatment of IFI	Oral, IV	Effective prophylaxis in AML; personalized dosing via CYP2C19 genotyping possible	Visual disturbances; CYP2C19 metabolism; need for therapeutic monitoring
Itraconazole	Prophylaxis and treatment of IFI	Oral capsule or solution, IV	Effective broad-spectrum prophylaxis and treatment of IFI, SUBA-ITZ	Hepatotoxicity, nausea, and gastrointestinal disturbances are common
Opelconazole	Investigational (pulmonary IFI)	Inhaled	Lung-specific delivery; reduced systemic toxicity	Early-stage trials; human safety data limited; no systemic coverage

Abbreviations: IFD = Invasive Fungal Disease; AML = Acute Myeloid Leukemia; MDS = Myelodysplastic Syndrome; post-HCT = Post-Hematopoietic Cell Transplantation; IV = Intravenous; IFI = Invasive Fungal Infection; QTc = Corrected QT Interval; SUBA-ITZ = super bio-available itraconazole.

## Data Availability

No new data were created or analyzed in this study. Data sharing is not applicable to this article.

## Abbreviations

The following abbreviations are used in this manuscript:

AML	Acute Myeloid Leukemia
ALL	Acute Lymphoblastic Leukemia
MDS	Myelodysplastic Syndrome
IFI	Invasive Fungal Infection
IFD	Invasive Fungal Disease
IV	Intravenous
HRCT	High-Resolution Computed Tomography
HIV	Human Immunodeficiency Virus
QTc	Corrected QT Interval
GVHD	Graft-versus-Host Disease
CYP3A4	Cytochrome P450 Family 3 Subfamily A Member 4
CYP2C19	Cytochrome P450 Family 2 Subfamily C Member 19
FDA	Food and Drug Administration
PC945	Investigational Inhaled Triazole (Opelconazole)
HCT	Hematopoietic Cell Transplantation
DSD	Drug Safety Data
